# Cardiovascular polypharmacy is not associated with unplanned hospitalisation: evidence from a retrospective cohort study

**DOI:** 10.1186/1471-2296-15-58

**Published:** 2014-03-31

**Authors:** Sarah C Appleton, Gary A Abel, Rupert A Payne

**Affiliations:** 1 Clinical School, University of Cambridge, Cambridge, UK; 2 Cambridge Centre for Health Services Research, Institute of Public Health, University of Cambridge, Cambridge, UK

**Keywords:** Cardiovascular, Polypharmacy, Primary care, Hospital admission

## Abstract

**Background:**

Polypharmacy is often considered suggestive of suboptimal prescribing, and is associated with adverse outcomes. It is particularly common in the context of cardiovascular disease, but it is unclear whether prescribing of multiple cardiovascular medicines, which may be entirely appropriate and consistent with clinical guidance, is associated with adverse outcome. The aim of this study was to assess the relationship between number of prescribed cardiovascular medicines and unplanned non-cardiovascular hospital admissions.

**Methods:**

A retrospective cohort analysis of 180,815 adult patients was conducted using Scottish primary care data linked to hospital discharge data. Patients were followed up for one year for the outcome of unplanned non-cardiovascular hospital admission. The association between number of prescribed cardiovascular medicines and hospitalisation was modelled using logistic regression, adjusting for key confounding factors including cardiovascular and non-cardiovascular morbidity and non-cardiovascular prescribing.

**Results:**

25.4% patients were prescribed ≥1 cardiovascular medicine, and 5.7% were prescribed ≥5. At least one unplanned non-cardiovascular admission was experienced by 4.2% of patients. Admissions were more common in patients receiving multiple cardiovascular medicines (6.4% of patients prescribed 5 or 6 cardiovascular medicines) compared with those prescribed none (3.5%). However, after adjusting for key confounders, cardiovascular prescribing was associated with fewer non-cardiovascular admissions (OR 0.66 for 5 or 6 vs. no cardiovascular medicines, 95% CI 0.57-0.75).

**Conclusions:**

We found no evidence that increasing numbers of cardiovascular medicines were associated with an increased risk of unplanned non-cardiovascular hospitalisation, following adjustment for confounding. Assumptions that polypharmacy is hazardous and represents poor care should be moderated in the context of cardiovascular disease.

## Background

Polypharmacy is widely considered as the prescribing of multiple medications, is often perceived to be inappropriate, and appears to be increasing in prevalence [1]. Although no single agreed definition exists for polypharmacy [2], and it may be thought of as either appropriate or problematic, the total number of medicines has nonetheless been identified as a patient characteristic associated with high risk prescribing [2]. A number of undesirable outcomes are associated with polypharmacy, including adverse drug events [3], increased mortality [4], poor adherence to treatment [5] and impaired quality of life [6]. Polypharmacy has also been found to be a strong predictor of preventable medicine-related hospital admissions [3], as well as unplanned hospitalisation more generally [4,5]. On-going care of patients with long-term conditions in the UK usually falls to the general practitioner (GP), often requiring coordinating the use of multiple medicines; the UK performance-related pay scheme, the Quality Outcomes Framework (QOF), recognises this in a requirement to undertake regular medication review for patients in receipt of multiple medicines.

Cardiovascular disease (CVD) is one area where polypharmacy may be of particular relevance. CVD is the leading cause of death globally [6], and associated polypharmacy is common, driven by numerous evidence-based guidelines advocating treatment with multiple therapeutic drug classes [7-9]. Indeed, in the UK cardiovascular medicines account for £1.35 billion annually, or 15% of the total primary care prescribing budget [10].

Despite the clear burden of cardiovascular disease and the volume of medications used in its management it remains unclear, however, whether prescribing of multiple cardiovascular medicines, which may well be entirely appropriate and in keeping with current evidence and guidance, is necessarily still associated with adverse outcomes. Polypharmacy is often considered simplistically as multiple medicines, irrespective of clinical appropriateness, and therefore we decided to examine whether this was proper. We decided to examine the potential impact of polypharmacy on unplanned hospital admissions, as a general measure of quality of care. Whereas routine hospital admissions or outpatient attendances may be considered appropriate measures in the management of long-term conditions, unplanned hospitalisation is generally undesirable. We hypothesised that increasing numbers of cardiovascular medicines might lead to unplanned hospital admission, through adversely impacting upon medication adherence, as well as contributing to medication errors, interactions and adverse events. We also considered that polypharmacy may reflect a failure to use medications in a rationale and effective manner, and as such may reflect poorer care more generally, again contributing to unplanned admissions. It was decided to specifically examine non-cardiovascular admissions, as we did not believe it would be possible to tease out any adverse or beneficial effects of cardiovascular medication from underlying cardiovascular disease severity.

The aim of this study was therefore to examine whether cardiovascular polypharmacy was associated with unplanned non-cardiovascular hospital admissions.

## Methods

We conducted a retrospective cohort study using data from forty GP surgeries involved in the Scottish Practice Team Information project. This is a programme providing information on national morbidity trends and primary care activity, and the surgeries involved are considered to be reasonably representative of the Scottish population as a whole [11]. Primary care data on patient demographic characteristics, diagnostic codes and electronic prescribing, were probabilistically linked to national Scottish acute hospital in-patient admissions records (the Scottish Morbidity Record). These data are considered of good quality during the relevant time period [12], and exclude accident and emergency department visits, and psychiatry and maternity admissions.

Patient details were based on data recorded as of 1 April 2006. All adults (aged ≥20 years) registered permanently with a surgery were included in the analysis. We recorded the presence of up to 40 long-term physical and mental health problems from the GP data. This list was developed by expert consensus, aiming to capture conditions significantly affecting quality of life, and those associated with high rates of morbidity or death. The list of conditions included a number of cardiovascular or cardiovascular-related problems: ischaemic heart disease, stroke (or transient ischaemic attack, TIA), cardiac failure, hypertension, atrial fibrillation and diabetes. Details of the development of this morbidity list are described elsewhere [13].

GP prescribing data was split into counts of cardiovascular and non-cardiovascular medicines, based on categorisation by the British National Formulary. We included prescriptions available on the index date for repeated issue to the patient (and issued at least once within the previous 12 months), in addition to recently issued one-off prescriptions (issued no earlier than the duration of prescription (usually 1-2 months) prior to the index date). Where more than one prescription of the same chemical entity was available, these prescriptions were counted only once; different chemical entities within the same drug class were counted separately. There is no consistent definition of polypharmacy, and treating the medication count as a simple binary factor has been shown to be unhelpful [14]. We therefore categorised the cardiovascular medicine count as none, 1 or 2, 3 or 4, 5 or 6, and 7 or more, and the non-cardiovascular medicine count as none, 1 to 3, 4 to 6, 7 to 9, and 10 or more, as a means of capturing the potentially non-linear nature of a medication count. Count categories were pragmatically chosen to provide 5 separate similarly-spaced groups for both cardiovascular and non-cardiovascular medicines. Because of the key, centralised position of general practice within the UK health system, we expect to have captured the majority of prescribing for long-term conditions, even when originally initiated in secondary care. We are, however, unable to account for over-the-counter treatments and short courses of treatment in hospital which have not been continued subsequently in the community.

All patients selected were followed up for one year from 1 April 2006 for the occurrence of at least one unplanned hospital admission. We included only admissions where the primary diagnosis was not a cardiovascular one (i.e. we excluded admissions with primary diagnosis ICD-10 codes I00 to I25), to avoid the potentially confounding problem of capturing admissions due to the condition for which the medication was being prescribed.

Based on the known distribution of cardiovascular prescribing, and assuming an admission rate of 4% in the baseline group (no drugs) and a stepwise relative change in admissions of 5% per increasing prescribing category, the study population provides over 90% power at the 5% significance level (two-sided) to detect an overall association between prescribing category and admission.

### Statistical analysis

Statistical analysis was performed using Stata v11.2 (StataCorp, Texas, USA). Mixed-effect binary logistic regression was used to model hospital admission for a primary non-cardiovascular problem, with count of cardiovascular medicines as the exposure of interest. Adjustment was made for sex, age group (20-year age ranges), an area-based socioeconomic deprivation measure (quintiles of the Scottish Index of Multiple Deprivation, SIMD), count of non-cardiovascular conditions, count of non-cardiovascular medicines, and the presence of certain cardiovascular conditions (ischaemic heart disease, heart failure, stroke including transient ischaemic attack, peripheral vascular disease, hypertension and atrial fibrillation). We also included a random effect for GP surgery to account for potential clustering of prescribing and admission characteristics by this factor.

One could argue that the effect of cardiovascular conditions amongst patients not receiving appropriate medicines may be different from that in those patients being treated. If so there may be some residual confounding in the model described above. In order to overcome this we conducted an additional more complex analysis, including interactions between the specific cardiovascular conditions and the cardiovascular medicine count. Exploratory analysis suggested that using interaction terms with the full 5-category drug count variable (as used in the non-interaction model) provided no further information than using a simple binary variable indicating presence or absence of any cardiovascular drug. We therefore present the results of a model using the more parsimonious parameterisation for the interaction term only (i.e. a model containing a single interaction between a binary variable indicating the presence of at least one cardiovascular drug and 6 binary variables representing the presence of each of the cardiovascular conditions – a total of 6 interaction terms).

We conducted a number of sensitivity analyses to check the robustness of our findings. We substituted cardiovascular medicine count in the main model with a count of eight different cardiovascular drug classes (ACE inhibitor or angiotensin receptor antagonist, beta-blocker, calcium channel blocker, diuretic, nitrate, anticoagulant or antiplatelet agent, lipid lowering drug, and any other cardiovascular medicine), to ensure that the means of classifying cardiovascular polypharmacy did not affect the results. To explore the impact of using an alternative approach to classifying cardiovascular morbidity, we included only the two commonest cardiovascular diseases (ischaemic heart disease and stroke) and the two commonest risk factors (hypertension and diabetes) as separate binary fixed effects, and included the other cardiovascular morbidities (heart failure, atrial fibrillation, peripheral vascular disease) in our “non-cardiovascular” morbidity count. Finally, to exclude the potential that excessive death might result in an apparent reduction in hospitalisation, we repeated the analysis excluding those with death within 6 months of admission (death data was unavailable for the full 12 months).

## Results

All 180,815 adult patients from the dataset were included in the analysis (Table 1). The study population had a median age of 49 years (inter-quartile range, IQR 36 to 63 years) and 49.3% of patients were male. The most commonly recorded cardiovascular conditions were hypertension (20.4%) and ischaemic heart disease (6.8%). Just under 60% of patients had at least one non-cardiovascular co-morbidity, with 5.3% having 5 or more. Slightly over a half of patients were prescribed at least one non-cardiovascular medicine, and 2.9% were prescribed 10 or more.

**Table 1:** Number of cardiovascular medications by different patient characteristics

		**Percentage of patients in each group on different numbers of cardiovascular drugs**				
	**Number of patients (% of total)**	**None**	**1 or 2**	**3 or 4**	**5 or 6**	**7 or more**
**All patients**	180815	74.6%	11.9%	7.8%	4.0%	1.7%
**Gender**						
Female	91739 (50.7%)	72.7%	14.1%	8.0%	3.7%	1.6%
Male	89076 (49.3%)	76.6%	9.7%	7.6%	4.2%	1.9%
**Age, years**						
20 to 39	55901 (30.9%)	97.7%	2.0%	0.3%	0.1%	0.02%
40 to 59	68525 (37.9%)	83.0%	10.7%	4.1%	1.5%	0.6%
60 to 79	45133 (25.0%)	46.1%	21.9%	17.9%	9.8%	4.3%
80 or more	11256 (6.2%)	23.4%	29.1%	26.8%	14.4%	6.2%
**Deprivation quintile**						
1, least deprived	31121 (17.2%)	78.8%	10.8%	6.5%	2.8%	1.0%
2	37261 (20.6%)	75.3%	12.1%	7.6%	3.6%	1.4%
3	45823 (25.3%)	73.6%	12.3%	8.1%	4.1%	1.8%
4	36098 (20.0%)	72.7%	12.4%	8.4%	4.4%	2.0%
5, most deprived	30512 (16.9%)	73.2%	11.8%	8.1%	4.7%	2.3%
**Cardiovascular condition**						
Hypertension	36859 (20.4%)	12.7%	35.9%	30.0%	14.9%	6.5%
IHD	12353 (6.8%)	4.8%	11.5%	31.1%	33.0%	19.6%
Stroke/TIA	5800 (3.2%)	10.9%	21.8%	32.6%	22.7%	12.0%
PVD	5473 (3.0%)	28.6%	21.7%	22.7%	17.1%	9.8%
Atrial fibrillation	3919 (2.2%)	7.8%	15.6%	27.7%	27.3%	21.6%
Heart failure	3673 (2.0%)	5.1%	14.2%	26.3%	29.6%	24.9%
**Non-cardiovascular comorbidity count**						
None	74230 (41.2%)	90.6%	5.3%	2.8%	1.1%	0.3%
1	44184 (24.4%)	77.3%	11.5%	7.0%	3.1%	1.1%
2	27199 (15.0%)	64.1%	16.6%	11.5%	5.6%	2.3%
3	16217 (9.0%)	53.1%	20.5%	14.7%	8.1%	3.6%
4	9357 (5.2%)	44.8%	23.5%	16.6%	10.2%	5.0%
5 or more	9628 (5.3%)	34.3%	26.3%	19.6%	12.5%	7.3%
**Non-cardiovascular drug count**						
None	87702 (48.5%)	89.8%	5.9%	3%	1%	0.3%
1 to 3	61015 (33.7%)	70.1%	14.4%	9.3%	4.5%	1.7%
4 to 6	19311 (10.7%)	47.4%	22.3%	16.4%	9.7%	4.2%
7 to 9	7549 (4.2%)	35.6%	24.7%	20.3%	12.5%	6.9%
10 or more	5238 (2.9%)	29.4%	27.6%	21.2%	13.2%	8.6%

IHD, ischaemic heart disease; TIA, transient ischaemic attack; PVD, peripheral vascular disease.

A quarter (25.4%) of patients were prescribed cardiovascular medicines, with 5.7% prescribed 5 or more (Table 1). Cardiovascular medicines were prescribed in greater numbers in older patients, the most socioeconomically deprived, those with greater numbers of non-cardiovascular co-morbidities and patients prescribed more non-cardiovascular medicines. With the exception of peripheral vascular disease, over half of patients with a specific cardiovascular condition were prescribed 3 or more cardiovascular medicines.

**Figure 1:**
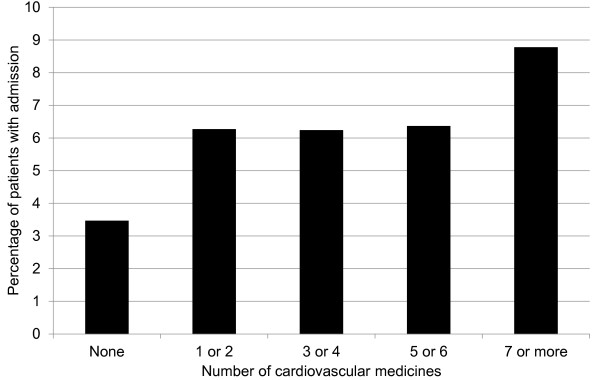
**Percentage of patients admitted to hospital.** Percentage of patients experiencing an unplanned non-cardiovascular hospital admission, for a given number of cardiovascular medicines.

A total of 7624 patients (4.2%) experienced at least one unplanned non-cardiovascular hospital admission. Admission was more common in older and more deprived patients, and in those with greater numbers of recorded non-cardiovascular morbidities and those prescribed higher numbers of non-cardiovascular medicines (Additional file 1: Table S1). Figure 1 shows how unplanned non-cardiovascular admissions were more frequently observed in those prescribed multiple (7 or more) cardiovascular medicines compared with those prescribed none (8.8% vs. 3.5%).

**Figure 2:**
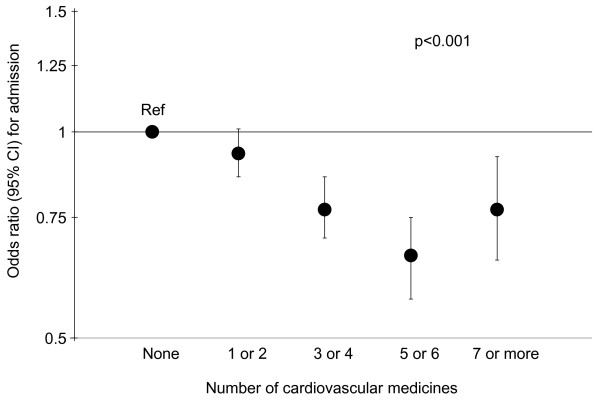
**Adjusted risk of unplanned admission.** Adjusted odds ratios (95% confidence intervals) of unplanned non-cardiovascular hospital admission for a given number of cardiovascular medicines.

The main multivariable logistic regression models are presented in Table 2, and show that male sex, and increasing age, socioeconomic deprivation, non-cardiovascular morbidity and numbers of non-cardiovascular medicines, were all independently associated with increased admissions. The presence of hypertension was associated with fewer unplanned admissions (OR 0.74, 95% CI 0.69-0.80). Other cardiovascular conditions were either not associated with hospitalisation, or showed modest positive associations with admission; the most marked effects were for stroke (OR for unplanned admission 1.81, 95% CI 1.65-1.99). There was strong evidence (p < 0.001) that prescribing of cardiovascular medicines was independently associated with a smaller risk of unplanned hospitalisation (in contrast to the positive association observed in the crude analysis); the strongest effect was observed for 5 or 6 cardiovascular medicines compared to none (OR 0.66, 95% CI 0.57-0.75). This is shown graphically in Figure 2.

**Table 2:** Adjusted logistic regression models for unplanned non-cardiovascular hospital admission

	**Adjusted odds ratio (95% CI) for unplanned admission**	**P value**
**Male**	1.24 (1.18-1.30)	<0.001
**Age, years**		
20 to 39	Ref	<0.001
40 to 59	0.84 (0.79-0.90)	
60 to 79	1.10 (1.02-1.19)	
80 or more	2.02 (1.84-2.22)	
**Deprivation quintile**		
1, least deprived	Ref	<0.001
2	1.11 (1.01-1.22)	
3	1.19 (1.09-1.30)	
4	1.28 (1.16-1.40)	
5, most deprived	1.46 (1.33-1.61)	
**Cardiovascular condition**		
Hypertension	0.74 (0.69-0.80)	<0.001
IHD	0.96 (0.87-1.06)	0.43
Stroke/TIA	1.81 (1.65-1.99)	<0.001
PVD	1.15 (1.04-1.28)	0.009
Atrial fibrillation	1.12 (0.99-1.27)	0.075
Heart failure	1.07 (0.95-1.22)	0.26
**Non-cardiovascular comorbidity count**		
None	Ref	<0.001
1	1.85 (1.71-2.00)	
2	2.74 (2.51-3.00)	
3	3.36 (3.05-3.71)	
4	4.16 (3.73-4.64)	
5 or more	4.70 (4.21-5.25)	
**Non-cardiovascular drug count**		
None	Ref	<0.001
1 to 3	1.15 (1.07-1.23)	
4 to 6	1.50 (1.37-1.64)	
7 to 9	1.91 (1.71-2.13)	
10 or more	2.85 (2.54-3.19)	
**Cardiovascular drug count**		
None	Ref	<0.001
1 or 2	0.93 (0.86-1.01)	
3 or 4	0.77 (0.70-0.86)	
5 or 6	0.66 (0.57-0.75)	
7 or more	0.77 (0.65-0.92)	

IHD, ischaemic heart disease; TIA, transient ischaemic attack; PVD, peripheral vascular disease; CI, confidence interval.

Results of the secondary analysis including an interaction effect between each cardiovascular condition and the presence or absence of at least one cardiovascular medicine are shown in Additional file 1: Table S2. As a result of including the interaction term, the effect of number of cardiovascular medicines relative to no cardiovascular medicines varies according to which cardiovascular conditions patients have. We instead present the odds ratios for a baseline of 1 or 2 medicines (and do not consider those patients taking no cardiovascular medicines) which is consistent across cardiovascular conditions due to the particular specification of the interaction term. Compared to the simple model, the effect size of cardiovascular medicine count was attenuated, although the negative association with hospitalisation persisted. This is because the effect of cardiovascular conditions amongst patients not taking cardiovascular medicines is generally greater than in those patients who are taking them (Additional file 1: Table S3).

The sensitivity analyses demonstrated that our results were robust to the definition of cardiovascular drug count used, the categorisation of long-term conditions, and the exclusion of deaths (data not shown).

## Discussion

This study examined the question of whether high levels of cardiovascular polypharmacy might be associated with adverse non-cardiovascular consequences, as measured by unplanned hospitalisation. Our study confirmed that cardiovascular polypharmacy is common, with 13.5% of all adult patients prescribed at three of more such medicines. Crudely, those patients taking more cardiovascular medicines are more likely to experience unplanned admissions. However, when adjustment is made for factors including non-cardiovascular morbidity and drug burden, we found no evidence of an increase in non-cardiovascular admissions with increasing numbers of cardiovascular medicines. Indeed, unplanned admissions were actually less likely to occur in the context of higher levels of cardiovascular prescribing, once other key factors had been adjusted for. This may reflect the fact that much of the prescribing of multiple cardiovascular medicines is driven by evidence-based guidelines, and as such reflects appropriate rather than problematic polypharmacy.

### Strengths and limitations

Our study has a number of important strengths. These include the large study population, the use of high quality data, and the linkage to secondary care data which helps improve capture of hospital events. There are, of course, a number of limitations. Firstly, we used a crude count of cardiovascular medicines, and did not take into account different drug classes or medication dose. However, our sensitivity analysis employed an alternative measure of polypharmacy based on drug classes and found similar results. Secondly, we were unable to factor in clinical indication for particular medicines. Nonetheless, we did include a number of cardiovascular diagnoses in our analysis (with an alternative approach used in the sensitivity analyses) including an interaction term in the secondary analysis. Thirdly, we used simple counts of non-cardiovascular long-term conditions and medicines, and it may be that results may differ if one looks at potential high-risk or complex groups, such as heart failure patients receiving non-steroidal anti-inflammatory drugs, or patients with severe renal impairment unable to tolerate ACE inhibitors or diuretics. More sophisticated measures, taking into account appropriateness of drugs and clinical context, should certainly be considered when evaluating polypharmacy, but to date consensus has not been reached around appropriate definitions, and one needs to balance the transparency and pragmatism of simple measures of polypharmacy against more complex approaches. Fourth, the analyses are based on data from 2006 to 2007, yet rates of medication use may have increased in the interim. It is possible that if this increase is also associated with more inappropriate use, then we may have underestimated the potential for adverse consequences. Finally, it could be argued that a more specific medicine-related outcome might be more relevant, such as hospital admission for adverse drug reactions. However, inappropriate polypharmacy can be considered to reflect poorer care more generally (e.g. non-adherence to clinical guidance, lack of continuity of care), and as such might be expected to manifest in broader measures of adverse outcome, of which unplanned hospitalisation is one. We specifically did not examine the association between cardiovascular medicines and cardiovascular admissions as we believe that this would be impossible to usefully interpret. For example, a positive association between the two may be due to patients on multiple cardiovascular medicines having poorer health, whereas a negative association may be due to more cardiovascular medicines reflecting better care; a combination of these effects might also be possible.

### Comparison with existing literature

Previous work has found associations between increasing unplanned hospitalisation and increasing degrees of polypharmacy [3-5]. However, this has not previously been examined in the specific context of cardiovascular medicines. Although non-cardiovascular admissions were observed more frequently in those receiving more cardiovascular medicines, this association was reversed once key confounding factors had been adjusted for. This negative association was attenuated once the interaction between cardiovascular disease and cardiovascular therapy was accounted for, suggesting some of the observed effect is driven by people with cardiovascular disease who are not receiving appropriate cardiovascular treatment, and are less well managed in general. Nonetheless, the effect still persisted to a lesser degree, and there are a number of potential explanations for this observation. Firstly, it is possible that more cardiovascular medicines may be associated with better clinical care generally. This might be due to better attentiveness to evidence-based practice for other clinical areas, that increased follow-up for cardiovascular disease provides an opportunity for clinicians to address non-cardiovascular morbidities, or that more frequent medication reviews are likely to occur with consequent optimisation of non-cardiovascular therapy. A second possibility is that more cardiovascular medicines may be associated with different patient behaviours; patients on these medicines may have a higher awareness of preventative strategies against admission and be more proactive about contacting their GP at other times. Finally, it may well be that some admissions are associated with cardiovascular disease, but are not coded as such [15], with examples including acute kidney injury, complications of diabetes, and smoking-related illness. It is seems unlikely that cardiovascular polypharmacy is primarily responsible for reducing non-cardiovascular admissions. It is also not possible to exclude the possibility that cardiovascular polypharmacy may be harmful in terms of unplanned admissions, but it would appear that overall any harm is more than outweighed by the associated better clinical care. It is of course possible that any such benefit would no longer be observed if there were improvements in holistic care not simply limited to those with specific long-term conditions such as cardiovascular disease. We can contrast the current findings with related work we have carried out examining the general case, where we found that increasing numbers of medicines was indeed associated with increased unplanned admissions, albeit an effect tempered in the context of increasing co-morbidity [5]. It is possible that our current findings differ from others’ work due to differences in the study populations and statistical models employed, although previous work has corrected for similar factors such as age, gender and comorbidity [3,4]. The differences between cardiovascular and general cases seem more likely to be accounted for by the more systematic and evidence-based nature of prescribing for cardiovascular disease, compared with medication use in other clinical areas. The current findings confirm that it is essential to consider the clinical context of polypharmacy, as well as providing clinicians with some confidence that the considerable burden of cardiovascular medications is in general not contributing to undesirable hospital admissions.

We corrected for a number of important confounding factors in our analysis. Older people were more likely to be admitted, reflecting the increased prevalence of long-term conditions and frailty. Socioeconomic deprivation was also associated with increased admissions. This is probably due to general higher levels of ill-health, and poorer resources in terms of coping strategies and support networks [16]. With the exception of stroke, cardiovascular morbidity was not particularly strongly associated with non-cardiovascular admissions. The weak associations observed may reflect general frailty, and the stronger effect found with stroke likely reflects complications of this condition less commonly found with other cardiovascular conditions, such as poor mobility and falls, or stasis pneumonia [17]. The negative association with hypertension has been noted previously, and could be suggestive of improved clinical monitoring in such patients [18]. It is worth noting that the effect of cardiovascular morbidity generally goes up in those patients not receiving any cardiovascular drugs. Non-cardiovascular morbidity and medicine count are both unsurprisingly associated with increased admissions. Medicine count may in this circumstance either reflect increasing disease severity, or illness not captured by the simple morbidity count. Of course, there are numerous other factors related to prescribing that we were unable to account for, beyond simply number of medications. These include physician adherence to clinical guidelines, patient experience of care, potential adverse effects and aspects of the doctor-patient relationship. Some of these issues might be addressed through the use of more sophisticated metrics of polypharmacy, and some may themselves be influenced by treatment burden; such factors certainly have the potential to modulate the effect of polypharmacy that we observed.

## Conclusions

This study has shown that cardiovascular polypharmacy is common and suggests that adverse consequences, as measured by unplanned hospital admissions, are not an inevitable consequence of prescribing multiple medicines. Crude measures of polypharmacy should therefore not be used as quality metrics or as predictors of hospital admission, and instead should take into account clinical context. Further work is merited to determine whether these observations vary between different cardiovascular conditions, or across different clinical areas where multiple prescribing is common.

### Ethical approval

This work was approved by the Privacy Advisory Committee of the Information Services Division of NHS National Services Scotland.

## Competing interests

The authors declared that they have no competing interests.

## Authors’ contributions

SA and RP conceived the study and conducted the analyses. GA advised on the statistical analysis. All authors contributed to interpretation of the findings. SA wrote the first draft of the manuscript, and all authors contributed to revision of the manuscript and approved the final manuscript.

## Pre-publication history

The pre-publication history for this paper can be accessed here:

http://www.biomedcentral.com/1471-2296/15/58/prepub

## Supplementary Material

Additional file 1: Table S1 Characteristics of admitted patients. **Table S2.** Effect of cardiovascular drug count, assessed by model with and without interaction terms between cardiovascular clinical conditions and treatment with one or more cardiovascular medicines. **Table S3.** Variation in effect of cardiovascular conditions with treatment or otherwise with cardiovascular medicines, based on model including interaction terms between cardiovascular clinical conditions and treatment with one or more cardiovascular medicines. Click here for file
