# Supplementary material for: Cardiovascular polypharmacy is not associated with unplanned hospitalisation: evidence from a retrospective cohort study
Source: BMC Fam Pract. 2014 Mar 31;15:58. doi: 10.1186/1471-2296-15-58 (PMC3997839; doi:10.1186/1471-2296-15-58)
Supplement: Additional file 1: Table S1 — Characteristics of admitted patients. Table S2. Effect of cardiovascular drug count, assessed by model with and without interaction terms between cardiovascular clinical conditions and treatment with one or more cardiovascular medicines. Table S3. Variation in effect of cardiovascular conditions with treatment or otherwise with cardiovascular medicines, based on model including interaction terms between cardiovascular clinical conditions and treatment with one or more cardiovascular medicines. [file 1471-2296-15-58-S1.docx]

**Additional file**

*Table S1: Characteristics of admitted patients*

|  | **Number of patients** | **% of all patients** | **Number of unplanned admissions (row %)** |
| --- | --- | --- | --- |
| **All patients** | 180815 | 100% | 7624 (4.2%) |
| **Gender** |  |  |  |
| Female | 91739 | 50.7% | 3973 (4.3%) |
| Male | 89076 | 49.3% | 3651 (4.1%) |
| **Age, years** |  |  |  |
| 20 to 39 | 55901 | 30.9% | 1747 (3.1%) |
| 40 to 59 | 68525 | 37.9% | 2173 (3.2%) |
| 60 to 79 | 45133 | 25% | 2379 (5.3%) |
| 80 or more | 11256 | 6.2% | 1325 (11.8%) |
| **Deprivation quintile** |  |  |  |
| 1, least deprived | 31121 | 17.2% | 908 (2.9%) |
| 2 | 37261 | 20.6% | 1376 (3.7%) |
| 3 | 45823 | 25.3% | 1867 (4.1%) |
| 4 | 36098 | 20% | 1726 (4.8%) |
| 5, most deprived | 30512 | 16.9% | 1747 (5.7%) |
| **Cardiovascular condition** |  |  |  |
| Hypertension | 36859 | 20.4% | 1995 (5.4%) |
| IHD | 12353 | 6.8% | 939 (7.6%) |
| Stroke/TIA | 5800 | 3.2% | 731 (12.6%) |
| PVD | 5473 | 3% | 470 (8.6%) |
| Atrial fibrillation | 3919 | 2.2% | 366 (9.3%) |
| Heart failure | 3673 | 2% | 390 (10.6%) |
| **Non- cardiovascular comorbidity count** |  |  |  |
| None | 74230 | 41.1% | 1258 (1.7%) |
| 1 | 44184 | 24.4% | 1454 (3.3%) |
| 2 | 27199 | 15% | 1442 (5.3%) |
| 3 | 16217 | 9% | 1194 (7.4%) |
| 4 | 9357 | 5.2% | 954 (10.2%) |
| 5 or more | 9628 | 5.3% | 1322 (13.7%) |
| **Non- cardiovascular drug count** |  |  |  |
| None | 87702 | 48.5% | 1979 (2.3%) |
| 1 to 3 | 61015 | 33.7% | 2447 (4.0%) |
| 4 to 6 | 19311 | 10.7% | 1478 (7.7%) |
| 7 to 9 | 7549 | 4.2% | 839 (11.1%) |
| 10 or more | 5238 | 2.9% | 881 (16.8%) |
| **Cardiovascular drug count** |  |  |  |
| None | 134914 | 74.6% | 4669 (3.5%) |
| 1 or 2 | 21585 | 11.9% | 1352 (6.3%) |
| 3 or 4 | 14087 | 7.8% | 878 (6.2%) |
| 5 or 6 | 7139 | 3.9% | 454 (6.4%) |
| 7 or more | 3090 | 1.7% | 271 (8.8%) |

*Table S2: Effect of cardiovascular drug count, assessed by model with and without interaction terms between cardiovascular clinical conditions and treatment with one or more cardiovascular medicines*

|  | **Odds ratio (95% CI) for unplanned admissions** | |
| --- | --- | --- |
| **Cardiovascular drug count** | Simple model | Interaction model* |
| 1 or 2 | Ref | Ref |
| 3 or 4 | 0.83 (0.76-0.91) | 0.88 (0.80-0.97) |
| 5 or 6 | 0.71 (0.62-0.80) | 0.77 (0.67-0.87) |
| 7 or more | 0.83 (0.70-0.98) | 0.92 (0.78-1.09) |

*CI, confidence interval*

* *p<0.001, joint-test of interaction terms (log-likelihood ratio test)*

*Table S3: Variation in effect of cardiovascular conditions with treatment or otherwise with cardiovascular medicines, based on model including interaction terms between cardiovascular clinical conditions and treatment with one or more cardiovascular medicines*

|  | **Odds ratio (95% CI) for unplanned admissions** | |
| --- | --- | --- |
| **Cardiovascular condition** | No cardiovascular medicines | 1 or more cardiovascular medicine |
| Hypertension | 0.91 (0.79-1.05) | 0.67 (0.62-0.73) |
| IHD | 1.49 (1.12-2.00) | 0.89 (0.80-0.98) |
| Stroke/TIA | 3.44 (2.77-4.28) | 1.60 (1.45-1.77) |
| PVD | 1.02 (0.81-1.27) | 1.19 (1.06-1.34) |
| Atrial fibrillation | 1.67 (1.13-2.47) | 1.06 (0.93-1.21) |
| Heart failure | 0.53 (0.29-0.97) | 1.13 (0.99-1.28) |

*CI, confidence interval; IHD, ischaemic heart disease; TIA, transient ischaemic attack; PVD, peripheral vascular disease*
